# OxInflammatory Responses in the Wound Healing Process: A Systematic Review

**DOI:** 10.3390/antiox13070823

**Published:** 2024-07-09

**Authors:** Fernanda Barbosa Lopes, Mariáurea Matias Sarandy, Rômulo Dias Novaes, Giuseppe Valacchi, Reggiani Vilela Gonçalves

**Affiliations:** 1 Department of General Biology, Federal University of Viçosa, Viçosa 36570-900, Minas Gerais, Brazil; 2 Plants for Human Health Institute, Animal Science Department, North Carolina State University, North Carolina Research Campus, Kannapolis, NC 28081, USA; 3 Department of Structural Biology, Federal University of Alfenas, Alfenas 37130-001, Minas Gerais, Brazil; 4 Department of Animal Biology, Federal University of Viçosa, Viçosa 36570-900, Minas Gerais, Brazil; 5 Department of Environmental and Prevention Sciences, University of Ferrara, 44121 Ferrara, Italy; 6 Department of Food and Nutrition, Kyung Hee University, Seoul 02447, Republic of Korea

**Keywords:** oxidative stress, inflammation, wound closure, inflammasome

## Abstract

Significant sums are spent every year to find effective treatments to control inflammation and speed up the repair of damaged skin. This study investigated the main mechanisms involved in the skin wound cure. Consequently, it offered guidance to develop new therapies to control OxInflammation and infection and decrease functional loss and cost issues. This systematic review was conducted using the PRISMA guidelines, with a structured search in the MEDLINE (PubMed), Scopus, and Web of Science databases, analyzing 23 original studies. Bias analysis and study quality were assessed using the SYRCLE tool (Prospero number is CRD262 936). Our results highlight the activation of membrane receptors (IFN-δ, TNF-α, toll-like) in phagocytes, especially macrophages, during early wound healing. The STAT1, IP3, and NF-kβ pathways are positively regulated, while Ca^2+^ mobilization correlates with ROS production and NLRP3 inflammasome activation. This pathway activation leads to the proteolytic cleavage of caspase-1, releasing IL-1β and IL-18, which are responsible for immune modulation and vasodilation. Mediators such as IL-1, iNOS, TNF-α, and TGF-β are released, influencing pro- and anti-inflammatory cascades, increasing ROS levels, and inducing the oxidation of lipids, proteins, and DNA. During healing, the respiratory burst depletes antioxidant defenses (SOD, CAT, GST), creating a pro-oxidative environment. The IFN-δ pathway, ROS production, and inflammatory markers establish a positive feedback loop, recruiting more polymorphonuclear cells and reinforcing the positive interaction between oxidative stress and inflammation. This process is crucial because, in the immune system, the vicious positive cycle between ROS, the oxidative environment, and, above all, the activation of the NLRP3 inflammasome inappropriately triggers hypoxia, increases ROS levels, activates pro-inflammatory cytokines and inhibits the antioxidant action and resolution of anti-inflammatory cytokines, contributing to the evolution of chronic inflammation and tissue damage.

## 1. Introduction

Skin wounds are a global public health challenge that imposes high costs on healthcare systems. Health organizations estimate that billions of dollars are spent each year to purchase preventative materials and treat wound complications [1,2]. Complex interactions between extracellular matrix molecules, soluble mediators, multiple resident cells, and infiltrating leukocyte subtypes surround the dynamic process of wound healing [3,4]. As the immediate goal of repair is to achieve tissue integrity and homeostasis, the first step is the inflammatory reaction. In this phase, vascular phenomena are characterized by hemostasis and coagulation, followed by cellular exudation [5]. During the proliferative phase, fibroblasts, keratinocytes, and endothelial cells proliferate to synthesize extracellular matrix (ECM) components, such as elastic fibers and collagen. During this phase, angiogenesis is stimulated to oxygenate and nourish the newly formed tissue, known as granulation tissue [3,4,6]. Granulation tissue is gradually replaced in the third phase (remodeling), and type III is replaced by type I collagen, increasing tissue resistance. Therefore, the third phase is characterized by fibroblasts, endothelial cell apoptosis, and progressive collagen accumulation (mainly type I fibers) [7,8,9,10].

The differences between acute inflammation and chronic inflammation are not only the duration but also the intensity of the cellular response. A low-intensity cellular response characterizes acute inflammation, whereas a high-intensity response characterizes chronic inflammation. When the damaged tissue is repaired and the infection is cleared, there is what is known as the ‘resolution of inflammation [11,12,13]. In the middle of acute and chronic inflammation is an OxInflammation process. To understand the relationship between oxidative stress and inflammation during the wound healing process, it is necessary to highlight cells as neutrophils, being responsible for producing immunological effectors such as interferon-γ (IFN-δ) and interleukins (IL), which will recruit macrophages to the wound area [14,15,16,17,18]. Through NF-kB and IL-12 stimulation, macrophages produce pro-inflammatory cytokines and growth factors, such as TNF-α, IL-6, and IL-8 [19,20], essential to resolve the inflammatory progression. In this process, Caspase-1 is autocatalytically activated upon inflammasome recruitment. Two key pro-inflammatory cytokines, pro-interleukin-1β (pro-IL-1β) and pro-interleukin-18 (pro-IL-18), which are initially inactive, have their activation facilitated by caspase-1 [21,22]. IL-1β induces the expression of genes that control vasodilatation, cell migration, and endothelial cell responses that facilitate immune cell infiltration into the injured tissue. IL-18 is required for interferon-gamma (IFN-δ) production and is a co-stimulatory cytokine involved with adaptive immunity [23]. However, these compounds produced and released by macrophages can contribute to tissue damage. The clinical consequences are persistent, low-grade, increased inflammatory response, impaired epithelialization, and granulation tissue formation. Therefore, it is necessary to have a perfect balance between pro-inflammatory and anti-inflammatory mechanisms, including anti-inflammatory cytokines and prostaglandin biosynthesis, to avoid poor tissue repair [24].

At the same time, phagocytes also produce reactive oxygen species (ROS) and nitrogen species (RNS), increasing their microbicide and tumoricidal activity. ROS and RNS are produced by macrophages in the process known as respiratory burst. These reactive species act as signaling molecules promoting the up-regulation of pro-inflammatory cytokines and down-regulation of anti-inflammatory cytokines, resulting in chronic inflammation. Although inflammation is essential to the healing process, an imbalance in the inflammatory process caused by oxidative stress can prolong the healing process because it is associated with an increase in ROS by activated immune cells, a deficit in angiogenesis and migration, and impaired cell proliferation [25,26,27]. In chronic inflammation, the tissue characteristically shows an infiltrate composed mainly of mononuclear cells (monocytes, macrophages, and lymphocytes), signs of angiogenesis and fibrosis [28]. Therefore, oxidative stress balance plays a fundamental role in resolving inflammation and healing skin wounds [29,30].

As part of the immune system, ROS are needed to kill bacteria and other microorganisms, consequently reducing inflammation [27]. In wound healing, nicotinamide adenine dinucleotide phosphate hydrogen (NADPH) oxidase reduces molecular oxygen and generates ROS excess in macrophages. Excessive ROS production inhibits cell migration and proliferation, affecting the expression and function of anti-inflammatory mediators. This effect enhances the inflammatory process, showing positive feedback among inflammatory and oxidative pathways [29]. Normal wound healing promotes the expression of many antioxidant genes [27,30], such as glutathione peroxidase (GPx), catalase (CAT), and superoxide dismutase (SOD), which from the antioxidant defense network in living systems and act at different levels [31,32], including limiting the excessive production of ROS, inhibiting the expression and activity of pro-inflammatory mediators such as COX-2 and iNOS, and attenuating the production of ROS [29,33]. However, the negative regulation of these antioxidant genes results in prolonged inflammation, and a delay in the healing process [34,35,36], and the excess ROS generated during inflammation can lead to cell damage, such as membrane rupture, DNA damage, and protein oxidation. By altering cellular functions, oxidative stress is induced, thus inhibiting cell migration and proliferation and affecting the expression and function of inflammatory mediators [37]. Without sufficient antioxidant activity, wound healing can be delayed, severe tissue damage can occur, and chronic inflammation can persist [38,39].

It is well known that inflammatory and oxidative markers play an essential role in skin wound closure. However, little is known about the relationship between inflammatory and oxidative effectors during different phases of the wound-healing process. A comprehensive analysis of signaling pathways and the relation of mechanisms involved in oxidative damage with its physiological response has not been systematically evaluated. Therefore, this study will help understand the mechanism underlying wound healing and guide decision-makers in developing new products and treatments that can accelerate skin wound closure. Consequently, it offered guidance to develop new therapies to control OxInflammation and infection and decrease functional loss and cost issues. The methodological bias analysis allows us to assess the strength of current evidence, findings, and research limitations in this field.

## 2. Material and Methods

### 2.1. Guiding Question

What is the central cellular mechanism’s relationship between inflammation and oxidative stress in a murine model of the skin wound healing process? What are the primary inflammatory and oxidative effectors involved with skin wound healing? Finally, what are the main inflammatory and oxidative mechanisms activated during skin wound healing?

### 2.2. Literary Research

**Figure 1:**
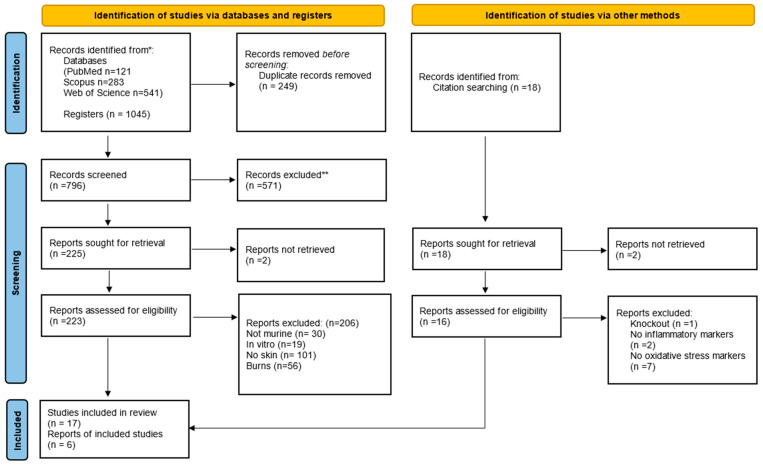
PRISMA (Preferred Reporting Items for Systematic Reviews and Meta-Analyses) flow diagram. The flowchart indicates the research records obtained at all standardized stages of the search process required to develop systematic reviews and meta-analyses. Based on the PRISMA statement (http://www.prisma-statement.org (accessed on 3 May 2024)). * Consider, if feasible to do so, reporting the number of records identified from each database or register searched (rather than the total number across all databases/registers). ** If automation tools were used, indicate how many records were excluded by a human and how many were excluded by automation tools.

This systematic review was conducted in accordance with the PRISMA guidelines (Figure 1) [40], ensuring thorough data selection, extraction, and analysis. A detailed literature search was conducted using the PubMed/Medline, Scopus, and Web of Science databases. On 13 April 2021, an advanced search was carried out on these platforms. The search strategy was designed to include two main approaches: direct searches in electronic databases and indirect screening of the reference lists of identified studies.

The search filters were built around four key terms: wound healing, oxidative stress, inflammation, and animal studies. For PubMed/Medline, the search filter used standardized descriptors from the MeSH hierarchical thesaurus, the MeSH, and TIAB commands to extract indexed articles and citations. These descriptors were adapted to meet the search requirements of the Web of Science (TS = descriptor) and Scopus (TITLE-ABS-KEY [descriptor]) databases. No chronological limits were imposed, and all original full-text studies published up to 2021 were included in the review. Two reviewers, FBL and MMS, carried out the initial literature search, removed duplicate articles, and selected titles and abstracts based on predefined eligibility criteria detailed in Table S1.

The full-text articles of potentially relevant studies were then independently assessed for eligibility by three reviewers: FBL, MMS, and RVG. Agreement between reviewers during data selection and extraction was measured using the kappa test, resulting in a kappa value of 0.937. Any inconsistencies were resolved through consultation between the reviewers.

### 2.3. Selection of Studies

In order to ensure the integrity and reliability of our review of the wound healing process, we implemented a rigorous selection and analysis methodology. Initially, two reviewers, FBL and MMS, undertook the data extraction independently in order to eliminate any potential bias. This independent analysis was of the utmost importance in maintaining objectivity, particularly during the data collection and selection phases. The focus of our review was on original experimental studies conducted in vivo, specifically using animal models, and published in English. Furthermore, only studies with full texts available were included. In order to further refine our selection process, we established clear eligibility criteria. Firstly, studies that directly investigated the healing of skin wounds. Secondly, studies that analyzed oxidative and inflammatory markers which are key in assessing the wound healing process. Finally, studies that involved excisional wounds in animal models ensure a consistent and relevant research context. Exclusion criteria were: (i) articles without full-text available; (ii) secondary studies (i.e., literature reviews, commentary, letters to the editor, and editorials); and (iii) studies not peer-reviewed or formally published in indexed journals.

### 2.4. Data Extraction

Three independent reviewers (FBL, MMS, and RVG) extracted the essential data, which were categorized into five descriptive levels: characteristics of the publication (author, year, and country), characteristics of the animal model (species, sex, age, and weight), intervention (control group, dose, frequency, route and time of intervention), main outcomes observed after treatment and secondary outcomes. In case of disagreement on the extracted data, two additional reviewers (RVG and RDN) participated in the discussion to resolve the issue.

### 2.5. Bias Analysis

The quality of the studies was assessed using the SYRCLE’s (Systematic Review Center for Experimentation with Laboratory Animals) risk of bias (RoB) tool [41], examining various methodological domains. The protocol for this systematic review has been registered in PROSPERO (262 936) and is available in full on the NIHR HTA program website (https://www.crd.york.ac.uk/prospero/, accessed on 5 July 2021). Selection bias assessed the generation of the random sequence, baseline characteristics, and allocation concealment to ensure equivalent groups at baseline. Performance bias focused on random assignment and blinding of caregivers and researchers to minimize conscious or subconscious bias. Detection bias analyzed the random assessment of outcomes and blinding to ensure objective and consistent measurement of outcomes. Attrition bias involves evaluating incomplete outcome data since high dropout rates can lead to biased results. Communication bias checked the selective communication of results to ensure that all planned results were communicated. Other biases considered ethics, the appropriateness of statistical methods, and work safety measures, increasing the credibility and replicability of the study. We used Cochrane’s Review Manager 5.3 (RoB 2.0) program to create a figure that visually presented the risk of bias in all the included studies. The items in the RoB tool were categorized and scored as “yes” (low risk of bias), “no” (high risk of bias), or “unclear” (not reported, making the risk uncertain). This exhaustive approach ensures that conclusions are based on solid and reliable data, contributing to more effective and ethical scientific research.

## 3. Results

### 3.1. Publication Characteristics

The initial search resulted in 121 studies in PubMed/Medline, 283 in Scopus, and 541 in the Web of Science database, totaling 1045 studies, of which 249 duplicates were excluded. After reading the titles and abstracts, another 571 irrelevant studies were excluded, and 225 studies were read in full. Only 23 studies fully met the inclusion criteria and were included in the systematic review (Figure 1). The studies were performed in India (39%); Brazil, United States of America, and Korea (13% each); Tunisia, Taiwan, Malaysia, Japan, and Indonesia (4.35% each). The ethical approval for the use of animals in the experimental design was specified in all studies.

### 3.2. Characteristics of the Animal Models

Considering the twenty-three studies in this review, 16 (69.6%) of them were performed with rats and 7 (30.4%) with mice. All studies used non-knockout mice as animal models and an untreated control group (100%). Animal sex was reported in 78% of the studies (52% used males, 17% used females, and 9% used males and females). Age was presented in 78% of the studies, ranging between 4 and 24 weeks, and 21.7% ignored this data. Animal weight was reported in 61% (n = 14) of the studies (Table S2). Rats’ weight ranged between 150 g and 310 g among the Wistar (43.5%), Charles-Foster albino, and Sprague–Dawley strains (13% each). Mice weight ranged between 18 g and 30 g among the C57BL/6 mice and ICR mice (8.7% each); Swiss albino, BALB/c, hairless SKH-1, CrljBgi, and CD-1 (4.37% each) (Table S2). The data evaluated in this review were extracted from control animals only to investigate the relationship between oxidative stress and inflammation in the normal healing process.

### 3.3. Excisional Wound Characteristics

Dorsal skin wounds were performed in 22 studies, with 61% of the studies with excision wounds and 39% with excision and incision wounds (Table S3). The methodology and specification of hygiene and asepsis before performing the wounds were neglected in 69.6% of the studies, and 30.4% (n = 7) reported the use of alcohol or saline solution or betadine as an antiseptic. Of the studies performed in rats (n = 16, 69.6%), four of them showed wounds with 500 mm^2^, three of them showed wounds between 50 and 300 mm^2^, four of them showed wounds between 10 and 20 mm^2^, and four of them showed 2–6 mm^2^ wounds. Only one study with rats neglected this information. On the other hand, in studies using mice (n = 7, 30.4%), three reported 10 mm^2^ wounds, and four showed a wound between 3.5 and 8 mm^2^. Only one study in mice neglected this information.

The experimental period for monitoring wound healing in rats was 8 to 15 days (n = 5, 21.7%) and 16 to 22 days (n = 9, 56.2%). Only two studies (n = 2, 8.7%) did not report this period in rats. In mice, the experimental period ranged from 3 to 7 days (n = 4, 17.4%) and from 8 to 14 days (n = 3, 13%), according to Table S3.

### 3.4. Main Biological Results

#### 3.4.1. Oxidative Stress

**Figure 2:**
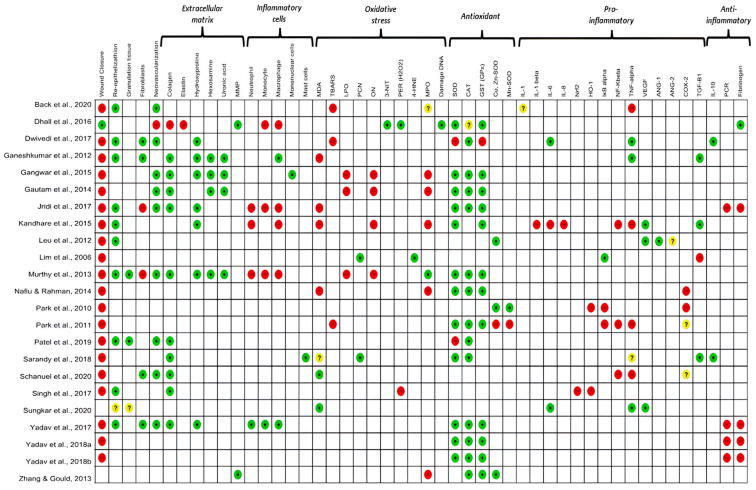
Results of the primary and secondary outcomes of the individual studies analyzed. The colour green: increased; red: decreased; yellow: undetermined and white: not analysed, indicate the results measured between the studies. MMP: matrix metalloproteinase; MDA: malondialdehyde; TBARS: Thiobarbituric acid reactive substances; LPO: lipid peroxidation; PCN: carbonylated protein; ON: nitric oxide; 3-NT: 3-nitrotyrosine; H2O2: hydrogen peroxide; 4HNE: 4-hydroxynonenal; MPO: metalloproteinase; SOD: superoxide dismutase; CAT: catalase; GST: glutathione transferase; CuZnSOD: copper–zinc superoxide dismutase; MnSOD: manganese superoxide dismutase; IL-1: interleukin-1; IL-1β: interleukin-1-beta; IL-6: interleukin-6; IL-8: interleukin-8; NRF2: nuclear factor erythroid factor 2; HO-1: the inducible isoform of HO; IKβ: Ikappaβ kinase; NF-kβ: nuclear factor kappa β; TNF-α: Tumor Necrosis Factor receptor alpha; VEGF: vascular endothelial growth factor; ANG1 and 2: angiotensin (1–2), COX-2: cyclooxygenase-2; TGF-β: transforming growth factor beta; IL-10: interleukin-10; CRP: C-reactive protein; FIB: fibrinogen. References of the articles in the figure: Back et al., 2020 [42]; Dhall et al., 2016 [43]; Dwivedi et al., 2017 [44]; Ganeshkumar et al., 2012 [45]; Gangwar et al., 2015 [46]; Gautam et al., 2014 [47]; Jridi et al., 2017 [48]; Kandhare et al., 2015 [49]; Leu et al., 2012 [50]; Lim et al., 2006 [51]; Murthy et al., 2013 [52]; Nafiu &Rahman, 2014 [53]; Park et al., 2010 [54]; Park et al., 2011 [55]; Patel et al., 2019 [56]; Sarandy et al., 2018 [57]; Schanuel et al., 2020 [58]; Singh et al., 2017 [59]; Sungkar et al., 2020 [60]; Yadav et al., 2017 [61]; Yadav et al., 2018a [62]; Yadav et al., 2018b [63]; Zhang & Gould, 2013 [64].

Oxidative stress was analyzed in all the studies included in this review. The level of antioxidant enzymes, such as catalase (CAT), glutathione (GSH) and superoxide dismutase (SOD), was analyzed in 74% of the studies. The pro-oxidant markers evaluated were malondialdehyde (30.4%; n = 7), lipid peroxidation (13%; n = 3), 3-nitrotyrosine (4.3%, n = 1), DNA damage (4.3%, n = 1), and 4-hydroxynonenal (4.3%, n = 1). In addition, the protein carbonyl (PCN) was analyzed in 8.7% of the studies. Among the oxidative stress mediators, hydrogen peroxide (H_2_O_2_) was investigated in 8.7% (n = 2) of the studies. Other markers, such as nitric oxide (NO) and myeloperoxidase (MPO), were analyzed in 17.4% (n = 4) and 30.4% (n = 7) of the studies, respectively (Figure 2).

#### 3.4.2. Inflammation

Most studies (78.3%) attempted to explain their results by quantifying different mediators and investigating inflammatory pathways activated during wound repair. The pro-inflammatory mediators evaluated were tumor necrosis factor-alpha (TNF-α) (n = 8, 30.8%), cyclooxygenase-2 (COX-2), transforming growth factor β (TGF-β) (n = 4, 14. 4%), interleukin 6 (IL-6), vascular endothelial growth factor (VEGF), nuclear factor- kβ (NF-κβ), IκB kinase (IKβ), and heme oxygenase 1 (HO-1) (13.4%, n = 3 each). Interleukin 1β (IL-1β), interleukin 8 (IL-8), NR-F2 related to the factor 2 (Nrf2), angiopoietin-1 and angiopoietin-2 (4.3%, n = 1 each) were additionally evaluated. Anti-inflammatory mediators such as interleukin-10 (IL-10) and C-reactive protein (CRP) were analyzed in 8.7% (n = 2) and 17.4% (n = 4) of the studies, respectively. Fibrinogen, a marker of the acute phase of inflammation, was analyzed in 22% (n = 6) of the studies (Figure 2).

#### 3.4.3. Relationship between Oxidative Stress and Inflammation

**Figure 3:**
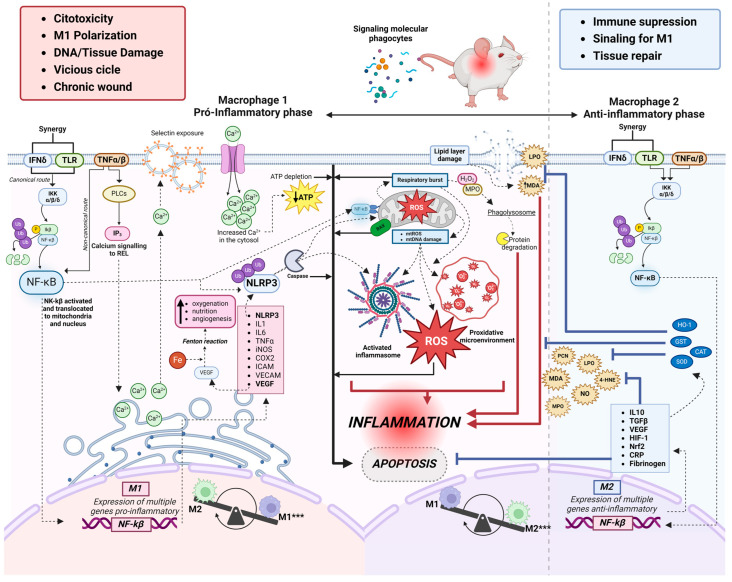
Overview of the interrelationship of major pathways and coexisting inflammatory mediators between oxidative stress and inflammatory process in excisional skin wound healing. *** Phenotypic plasticity of macrophages; presence of M1 (pro-inflammatory phase) and presence of M2 (anti-inflammatory phase). 4HNE: 4-hidroxinonenal; Ca^2+^: ion calcium; CAT: catalase; COX-2: cyclooxygenase-2; Fe^+^: ion iron; GST: glutathione transferase; H_2_O_2_: hydrogen peroxide; HIF-1: Hypoxia-inducible factor 1; ICAM: intercellular adhesion molecule; IFN-δ: Interferon-gamma receptor; IKK: inhibitor complex nuclear factor-κβ kinase; IKβ: IkappaB kinase; IL-1: interleukin 1; IL-6: interleukin-6; IL-10: interleukin 10; iNOS: Inducible nitric oxide synthase; IP3: IP3 signaling pathway; LPO: lipid peroxidation; MPO: metalloproteinase; NF-kβ: nuclear factor kappa β; NLRP3: inflammasome NLRP3; ON: nitric oxide; PCN: carboniled protein; PCR: C-reactive protein; ROS: reactive oxygen species; SOD: superoxide dismutase; TGF-β: transforming growth factor beta; TLR: toll-like receptor; TNF-α: Tumor Necrosis Factor receptor alpha; VECAM: vascular adhesion molecule; VEGF: vascular endothelial growth factor. Figure created on BioRender.com.

The oxidative markers investigated in this review were produced by polymorphonuclear cells (n = 4, 17.4%) and mononuclear cells (n = 7, 30%) during the inflammatory and proliferative phases of the skin wound healing process. There was a predominance of monocytes, neutrophils, and macrophages in the initial phase of inflammation up to 10 days after excision. There was a predominance of macrophages and neutrophils (n = 2, 28.5% each) in the initial phase of inflammation from 3 to 7 days, (n = 3, 43% each) from 10 to 12 days and (n = 2, 28.5% each) from 18 to 20 days after excision in the seven studies that evaluated cellularity (Table S3). We observed considerable evidence that macrophages are functionally polarised in response to pathogen-associated patterns (PAMPs) and damage-associated molecular patterns (DAMPs). Predominantly pro-inflammatory Macrophages (M1-type phenotypes) have a prolonged polarisation that may lead to tissue damage and contribute to pathogenesis. However, with an anti-inflammatory action (M2-type phenotypes), they play a critical role in resolving inflammation by producing anti-inflammatory mediators (Figure 2 and Figure 3).

#### 3.4.4. Classical Macrophages (M1)

Individual studies in this review describe the activation of IFN-δ and Toll-like receptors, which act synergistically and simultaneously with the TNFα/β pathway, initiating a pro-inflammatory cascade within the cytosol upon exposure to DAMPS and PAMPs. NF-κB activation occurs mainly through the phosphorylation of inhibitory molecules mediated by IκB (kinase (IKK) (26%), including IκBα [22,30,31,32,33,34]. Once activated, NF-kβ can translocate to the nucleus and contribute to the transcription of pro-inflammatory cytokines. In addition, it can also directly interact with the mitochondria and collaborate with NLRP3 (as described below). Our results showed that, during the inflammatory phase, the transcription of pro-inflammatory molecules such as IL-1 (4.3%) [42] and IL-6 (13.4%) [44,49,50,60] is responsible for activating cell migration and upregulates cytokines release such as VEGF (13.4%) [45,50,60], which promote tissue oxygenation. In addition, free iron availability increases, creating a pro-oxidative microenvironment and indirectly inducing angiogenesis. Furthermore, the studies analyzed showed increased levels of the B-cell inhibitor alpha gene, nuclear factor kappa light polypeptide enhancer (IκBα), and TNF-α in the inflammatory phase in 7 studies (34%) [42,44,45,49,55,58,60].

In parallel, the high expression of NLRP3 leads to the proteolytic cleavage of caspase-1, which is responsible for IL-1b and IL-18 maturation produced in response to TLR/NF-kβ pathway activation. The NLRP3 component of the inflammasome, now activated, goes on to be translocated for interaction with mitochondria, but its main role is the activation of Caspase1. The release of mitochondrial factors into the cytosol (mitochondrial ROS and DNA), together with NF-kβ, increases the activation of inflammasomes, the generation of ROS, and the formation of a pro-oxidant microenvironment. Another mitochondrial event is the respiratory burst, which generates oxidation products (H_2_O_2_ and MPO) and acidification of the phagolysosome, which are rapidly marked in polymorphonuclear cells and completely degrade cytosolic proteins, culminating in an inflammatory process. Our results showed that in one study [49] (4.3%), by quantifying polymerase gamma (pol-γ) and the BAX protein associated with Bcl-2, it occurs through apoptosis stimulation and inhibition of collagen-1 expression during NLRP3 inflammasome activation. All these events have positive feedback, further increasing inflammation and apoptosis. In addition, the studies included in this review indicated that a TNF-α-stimulated pro-inflammatory environment activates IP3 (13.4%) [44,45,61] and phospholipases (PLCs) pathways, which signal the release of calcium from the smooth endoplasmic reticulum (SER). IP3 triggers several stimuli, including calcium influx, mitochondrial ROS generation, and NLRP3 inflammasome activation. As a result, caspase-1, IL-1β, and IL-18 can be activated.

#### 3.4.5. Alternative Macrophages (M2)

Compensatory plasticity for alternatively activated macrophages (M2) occurs in response to inflammatory events. The response is induced by the synergistic activation of IFN-δ and Toll-like receptors and concomitant TNFα/β, which act in the same way as classical macrophages (M1), but now with a genetic expression of anti-inflammatory cytokines such as transforming growth factor-β1 (TGF-β1) (14.4%) [45,49,51,57], and IL-10 in 3 studies (13%). In addition, C-reactive protein and fibrinogen down-regulation was observed, indicating the end of the acute phase of inflammation in studies [48,61,62,63] (17.4%).

In two studies, metalloproteinase increased (8.7%) in the initial phase of inflammation and decreased rapidly after 7–10 days. This result may have occurred because tissue matrix metalloproteinase (TIMP) inhibitors play a crucial role in maintaining the balance of active and inactive MMPs during the late phase of the wound-healing process. Oxidation and the formation of carbonylated proteins are often associated with inflammatory processes. Metalloproteinases may regulate inflammation, and carbonylated proteins may result from the oxidative stress associated with inflammation. Only two studies [51,57] (8.7%) used the oxidation of carbonylated proteins as a marker of oxidative stress, which showed high levels, indicating a relevant antioxidant role.

Dynamic balance is demonstrated with reduced levels of TNF-alpha, IL-1beta, IL-6, IL-8, HO-1, Nrf2, IκBα, and NF-κβ in 6 studies [42,49,54,55,58,59]. These results show the final part of the activation of survival pathways, such as the NF-kβ pathway, which induces the expression of genes and other pro-inflammatory molecules critical to initiating the inflammatory process. Physiologically, the NF-κβ pathway stimulates the anti-inflammatory defenses of cells and reduces the harmful effects of oxidative stress by preventing premature and excessive activation of the NLRP3 inflammasome in macrophages [65]. In addition, it promotes autophagy [66], negatively regulates the activation of the NLRP3 inflammasome [67,68,69], and mitophagy, when associated with the p62 protein, provides an essential regulatory cycle through which NF-κβ orchestrates a reparative inflammatory response and avoids excessive collateral damage [70]. These results were observed over an average of up to 22 days of wound healing (Table S3). Most of the studies included in this review described an increase in the antioxidant enzymes CAT (55.9%) [44,46,47,48,52,53,55,56,57,61,62,63,64] and GST (51.6%) [43,46,47,48,49,52,53,55,61,62,63,64] to quench H_2_O_2_ (8.6%) [43,59] and prevent ROS accumulation in the injury site. As a result, anti-apoptotic signaling pathways are activated. This stimulates cells’ survival and proliferation.

Inflammatory cells (neutrophils, macrophages, and mast cells) are described in Figure 2, whose primary function is to eliminate potential microorganisms [71] and to induce tissue repair [43,45,46,48,49,52,57,61], were assessed in 35% of the studies. With the regional presence of mononuclear cells [45,46,57,61] and the production and release of chemical mediators produced by them, fibroblast migration and activation are intensified. Fibroblasts are the main constituents of granulation tissue. Under the influence of growth factors, they are activated and migrate from the edges to the wound’s center.

As a result, morphological changes occur in the scar tissue. The increase in re-epithelialization (43.5%) [42,44,45,48,49,50,52,56,59,61], granulation tissue rich in fibroblasts (17.4%) [44,45,58,61], blood vessels (39%) [42,44,46,47,48,52,56,58,61] and extracellular matrix components such as collagen (43.5%) [48,49,50,52,53,57,64,65,66,67], hydroxyproline (30.4%) [44,45,46,48,49,52,61], hexosamine and uronic acid (7.4% each) [45,46,47,52] were observed in association with increased antioxidant enzymes activity and progression of resolutive inflammatory phase.

At the end of the inflammatory process, there is a gradual decrease in pro-inflammatory and an increase in anti-inflammatory mechanisms. The reduced inflammation may be related to the reduction in NF-kβ activation and Ca^2+^ mobilization, which inactivates the NLRP3 inflammasome. The events that occur in the anti-inflammatory phase lead to inhibition of apoptosis, immunosuppression, M1 Signaling, inflammation resolution, and tissue repair. All relevant results, including histological data and oxidative stress markers, antioxidants, and cytokines, are described in Figure 3.

#### 3.4.6. Clinical Perspectives

The treatment of skin wounds is influenced by various clinical factors, including the size, depth, and location of the injury. A detailed understanding of the mechanisms activated during wound healing is crucial in designing appropriate treatment strategies to minimize the risk of scarring and infection. Despite the availability of several treatment methods, such as the physical removal of debris and biofilm and the application of systemic and topical antimicrobials, substantial progress is still required. One promising area of research involves the controlled release of antimicrobial agents through tissue-engineered scaffolds. However, progress in this field is hampered by a limited understanding of the primary mechanisms activated during tissue recovery. These mechanisms are complex and involve a series of biological processes that need to be thoroughly understood to enhance treatment efficacy. The reconstruction of wounds using auto- and allografts is a common practice for tissue replacement. However, these methods have significant limitations and often demonstrate low clinical value in cases of delayed skin wound healing. One reason for this fragility is that most research in this area has been restricted to in vitro tests, which do not replicate the complexities of living organisms. In this context, this review allows an understanding of the OxInflammation process during the wound healing process based on the studies that used in vivo models. It was possible to observe that many therapies are in preliminary stages and need to be understood more about the mechanisms before being translated into the human context. Therefore, OxInflammation is an important mechanism that should be controlled to solve clinical problems associated with skin wound healing.

### 3.5. Risk of Bias and Methodological Quality Assessments

**Figure 4:**
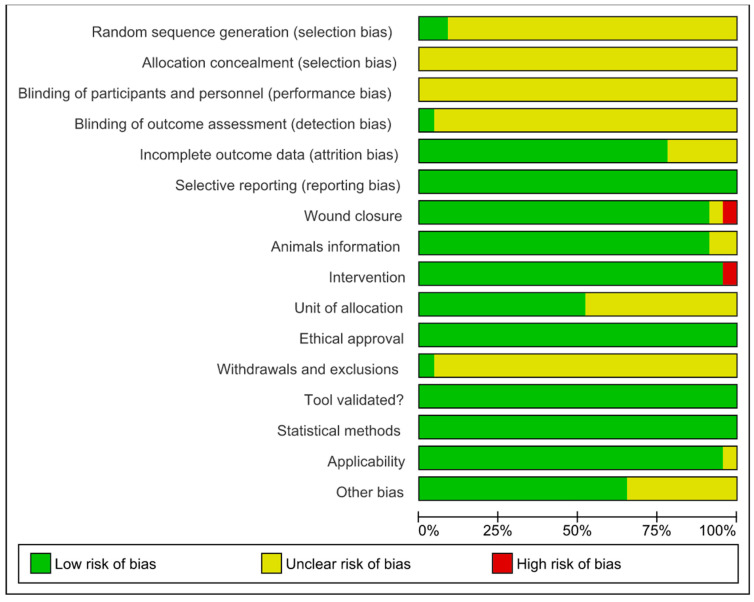
Risk of bias and methodological quality indicators for all studies included in the systematic review that assessed inflammation and oxidative stress during skin wound healing.

**Figure 5:**
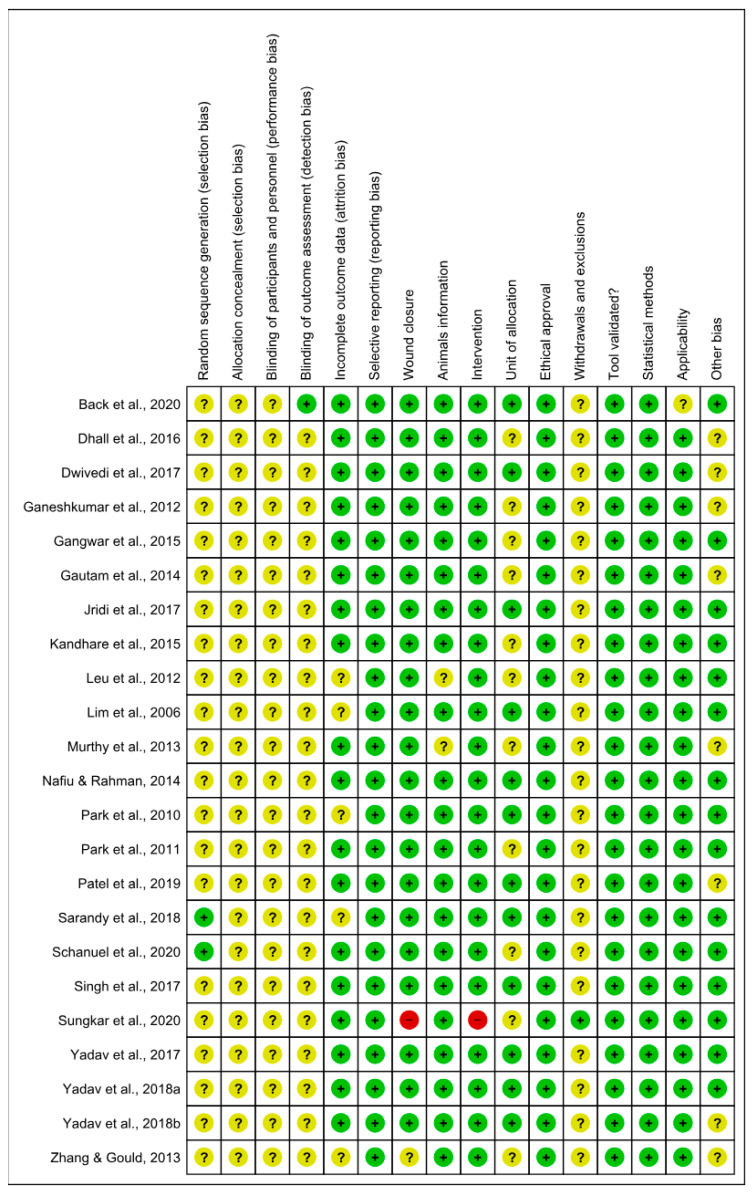
Risk of bias summary: review authors’ judgments about the risk of bias items for each included study. Green: low risk of bias; Yellow: unclear risk of bias; and Red: high risk of bias. References of the articles in the figure: References of the articles in the figure: Back et al., 2020 [42]; Dhall et al., 2016 [43]; Dwivedi et al., 2017 [44]; Ganeshkumar et al., 2012 [45]; Gangwar et al., 2015 [46]; Gautam et al., 2014 [47]; Jridi et al., 2017 [48]; Kandhare et al., 2015 [49]; Leu et al., 2012 [50]; Lim et al., 2006 [51]; Murthy et al., 2013 [52]; Nafiu &Rahman, 2014 [53]; Park et al., 2010 [54]; Park et al., 2011 [55]; Patel et al., 2019 [56]; Sarandy et al., 2018 [57]; Schanuel et al., 2020 [58]; Singh et al., 2017 [59]; Sungkar et al., 2020 [60]; Yadav et al., 2017 [61]; Yadav et al., 2018a [62]; Yadav et al., 2018b [63]; Zhang & Gould, 2013 [64].

The risk of bias analyzed from SYRCLE’s tool is described in Figure 4 and Figure 5. The results obtained from individual studies are reported in Figure 3. None of the trials met all the methodological criteria investigated. However, the number of studies with an overall high risk of bias was low (4.3%) [41]. Only two studies provided clear information on the generation of the random sequence [58,61], allocation concealment and blinding of participants were not reported in 100% of the studies, and blinding of results was only described in one study [42]. However, a low risk of bias was found when assessing whether the studies had incomplete data on outcomes (78.3%) [42,43,44,45,46,47,48,49,52,53,55,56,58,59,60,61,62,63], wound closure (91.3%) [42,44,45,46,47,48,49,50,51,52,53,54,55,56,57,58,59,61,62,63], intervention (95.6%) [42,43,44,45,46,47,48,49,50,51,52,53,55,56,57,58,59,61,62,63,64], selective reporting, ethical approval, validation tool and statistical methods (100% each) in studies, applicability (95.6%) [42,43,44,45,46,47,48,49,50,51,52,53,55,56,57,58,59,61,62,63,64] and other sources of bias (65.2%) [42,44,46,48,49,50,51,53,55,57,58,59,60,62,63]. In addition, the current evidence is reliable because the bias analysis showed a low risk of bias. Some methodological quality indicators showed an unclear and limited risk of bias due to underreporting and guideline adherence. A single study was classified as high risk of bias because it did not report wound closure and intervention, essential indicators in studies involved in wound healing.

## 4. Discussion

### 4.1. General Characteristics of the Studies

In this study, we conducted a systematic review to investigate the relationship between inflammation and oxidative stress in the skin wound healing process in a mouse model. Our results provided strong evidence that inflammation and oxidative stress are coexisting processes with a clear overlap in pathways and mechanisms. Furthermore, the morphological changes observed in the skin repair were mainly associated with antioxidant, vascular, chemotactic, and survival activation effectors such as NF-Kβ, IKβ, IP3, and IFN-δ. Mice and rats were the most commonly used animal models, possibly due to their greater availability, low cost, and ease of use. These characteristics can justify their primary choice in half of the studies distributed in different continents analyzed, with India being the country that presented the most studies. Indian traditional medicine is one of the oldest medical sciences in the world [72], being the largest producer of medicinal plants. In addition, about 70% of the rural population depends on the traditional system based on herbal medicine [73,74].

In addition, prolonged wound healing can result in more damaged cells, chronic inflammation, and high levels of ROS, which can compromise the effectiveness of the repair process and the overall health of the affected area [43,58]. It is, therefore, important to promote effective and rapid healing whenever possible to minimize these adverse effects [56].

### 4.2. Relationship between Inflammation and Oxidative Stress

It is already known that inflammation and oxidative stress coexist [75,76,77,78,79] in a mechanism defined as “OxInflammation” [13], which contributes to prolonged inflammation in diabetes, obesity, and burns [80,81,82,83]. However, the different pathways, mechanisms, and molecules that link these processes have not been studied together (Figure 3).

In particular, macrophages have a plasticity that allows them to respond to changes in their microenvironment. Accordingly, these cells are able to change their activation phenotype, leading to the broad classification of classical (M1) or alternative (M2) macrophage activation, which is present as infection progresses and cells are damaged [84]. Initially, the IP3 pathway is stimulated by pro-inflammatory cytokines such as TNF-α and IL-1 [85,86], which act directly on the smooth endoplasmic reticulum, stimulating intracellular Ca^2+^ release and enabling the release of adhesion molecules (selectins) present in Weibel-Palade vesicles. These adhesion molecules facilitate diapedesis [85]. During this process, neutrophils are the first cell type to arrive at the injured site, followed by macrophages [84]. In addition to the IP3 pathway, pro-inflammatory cytokines (TNF-α or IL-1) activate the NF-Kβ and IKK (survival) pathways via membrane receptors and induce the expression of other pro-inflammatory genes (IL-1, IL-18, TNF-α, iNOS, COX-2) [50,86,87,88]. At inflammation sites, activated inflammatory cells release free radicals [77], as well as enzymes and chemical mediators, resulting in molecular damage (DNA, proteins, and lipids) and oxidative stress that must be eliminated. Toll-like receptors are activated by PAMPs to trigger a pro-inflammatory response, triggering transcription factors, pro-inflammatory gene expression, and ROS biosynthesis [78]. Next, one of the key events required for efficient tissue repair is apoptotic cell elimination by phagocytes in the injured tissue [59]. The abundant infiltrating neutrophils represent a large reservoir of short-lived inflammatory cells programmed to undergo apoptosis [56]. Another example related to the link between oxidative stress and apoptosis is represented by the release from the mitochondria of ROS mediators that are quickly recognized by immune cells and promote their migration [4,28]. ROS (pro-oxidative microenvironment, mtROS, ROS, and inflammasomes) produced as part of the inflammatory response facilitate the clearance of toxins and pathogens and induce antioxidant gene expression. However, prolonged cytokines production can stimulate oxidative stress and chronic inflammation-related diseases and eventually lead to piroptosis [28,75,76]. In this oxidative environment, the overexpression of TGF-β has been associated with an increase in the deposition of extracellular matrix and the activation of fibroblasts [89,90]. Another important cytokine in this phase of acute inflammation is IFN-δ. In synergy with tool-like receptors or membrane lipopolysaccharide (LPS), IFN-δ can induce NF-κB activation and M1 macrophages-related immunological effectors such as IL-6, TNF-α, IL-1β and nitric oxide (NO) [76]. The imbalance in cytokine production, such as IL-1, TNF-α, and IL-6, activates signaling pathways that increase ROS production, including superoxide and hydrogen peroxide [91,92], during inflammatory responses and activates the IFN-δ pathway, which will synergistically increase ROS production via mitochondrial ROS and ROS via NADPH oxidase [92,93]. The main function of this pro-inflammatory cytokine is to recruit leukocytes to an infection site and stimulate M1 macrophage polarization, which produces pro-inflammatory cytokines such as IL-1 and IL-12 [85]. Interestingly, IFN-δ increases NO production by macrophages [45,88]. During the inflammatory process, NO is produced by inducible NO synthase (iNOS) in a calcium-independent process [94,95,96]. It is also known that a mitochondrial form of NOS (mtNOS) exists, although detailed information about it is not yet fully understood [79,96]. NO is produced at low concentrations, has a short lifetime, and can generate reactive nitrogen species (RNS) such as nitrous acid (HNO_2_), nitrogen dioxide (NO_2_), and peroxynitrite (ONOO^−^), which results from NO reaction with superoxide ion (O_2_), an important oxidative stress initiator. In addition, Ahmed and Ismail [97] and Wani et al. [98] reported that the membrane receptors for IFN-δ trigger pathways that act in the cytosol of the macrophage, signaling the mitogen-activated protein kinase (MAPK)/c-Jun amino-terminal kinase (JNK) and producing inflammatory and apoptotic mediators that amplify the production of ROS and the inflammatory response. Carbonylated proteins are formed as a result of free radical attack on amino acid side chains [61]. Free radicals have a dual function: they positively stimulate macrophages to generate excessive oxidative stress to eliminate pathogens, induce cell death through caspase activation, and create an imbalance in glutathione levels [99]. Antagonistically, oxidative stress is activated in the presence of inflammation. The balance between ROS production and antioxidant defenses is important for the resolution of inflammatory diseases, as well as for efficient tissue repair [37]. In response to tissue injury, the body initiates a chemical signaling cascade (PRRs, DAMPs, PAMPs) that stimulates responses aimed at healing the injured tissue [18]. Low ROS levels activate signaling pathways to initiate physiological processes, whereas high levels damage biomolecules [37]. Signaling centers in cellular physiology, such as mitochondria, play a role in inflammatory diseases. Studies suggest that ATP synthase inhibition also directs a tissue homeostatic response by activating the NF-Kβ pathway (a physiological regulator of mitochondrial respiration)—through specific NF-Kβ receptors on the mitochondrial surface [100]. This process leads to the concomitant production of mtROS [101,102,103,104], which regulates cellular and tissue processes during healing [105]. This product activates the inflammasome NLRP3, which triggers pro-inflammatory interleukins such as IL-1β and IL-18 [21,106,107], an essential step in the innate immune response. Furthermore, under hypoxic conditions, NO can also be produced during the respiratory chain reaction [96,98,108]. The latter reactive nitrogen species (RNS) can further lead to ROS production, such as reactive aldehydes, malondialdehyde, and 4-hydroxynonenal [86,96]. During injury, there is a reduction in oxygen and ATP and an increase in ROS through the superoxide radical (O^2−^), hydrogen peroxide (H_2_O_2_), singlet oxygen [(O_2_ (1 Δg)], ozone (O_3_), nitric oxide (NO) and peroxynitrite anion (ONOO^−^) produced by the cell during the respiratory process (respiratory burst). Although they are physiologically produced by normal metabolic pathways, ROS are amplified during the inflammatory process, causing increased oxidative stress [29,109,110]. In addition, they induce the release of latent TGF-β, and chronic inflammation perpetuates its activation, creating a vicious cycle that leads to abnormal healing and the formation of hypertrophic scars [111,112,113]. The resulting cellular damage can be summarized in three ways: lipid peroxidation, protein degradation, and DNA damage [114]. The lipid membrane is highly susceptible to oxidative damage, setting off a chain reaction that not only impairs intramembrane transport but also generates toxic by-products. In addition, lipid peroxidation stimulates the production and action of pro-inflammatory mediators that increase ROS biosynthesis, leading to antioxidant depletion [115]. In this context, anti-inflammatory mediators have been observed to play an important role in redox signaling during skin wound healing, in addition to resolving the inflammatory process. Various endogenous and/or exogenous antioxidant defenses (CAT, SOD, GSH, and vitamin E) are present in tissues to minimize the toxicity of oxygen metabolites [78,79]. With redox balance, TGF-β inhibits the production of pro-inflammatory cytokines such as TNF-α, IL-1β, and IL-6, stimulates IL-10 [91], allows the differentiation of fibroblasts into myofibroblasts to help cover wounds [114], inhibits the TGF-β/Smad pathway and consequently reduces fibrosis and keloids [111,112].

**Figure 6:**
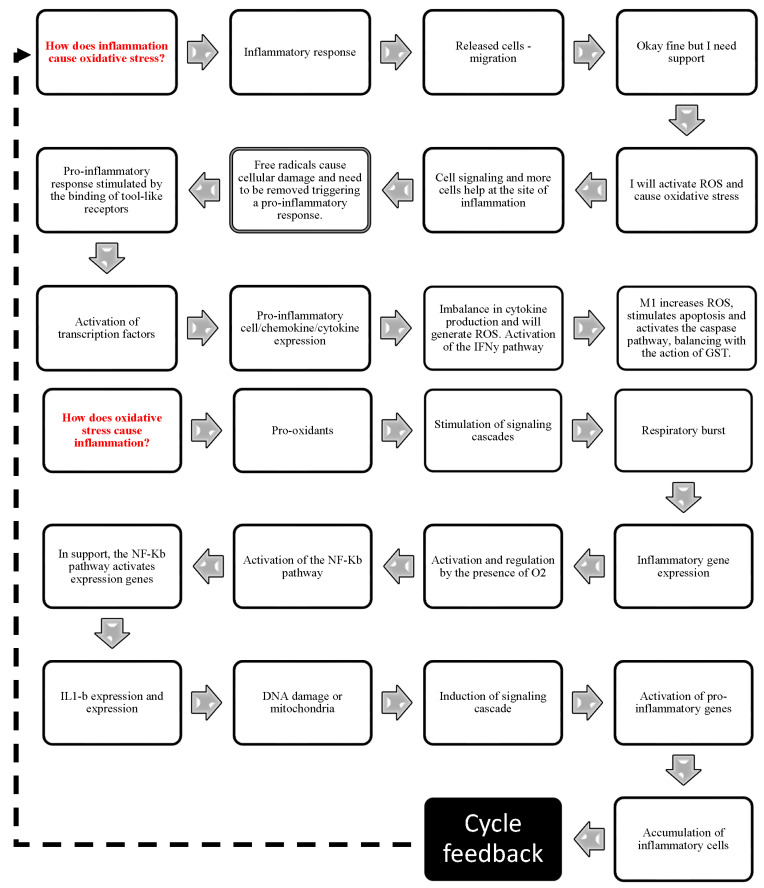
Continuous cycle and interrelating oxidative stress and inflammation, schematizing the major signaling pathways, synthesis of pro- and anti-inflammatory mediators, and antioxidant enzymes involved in the repair of excisional wounds.

Enzymes that protect cells exposed to ROS, such as cyclooxygenases (COX), myeloperoxidases (MPO), uncoupled nitric oxide synthase (NOS), peroxidases and NADPH oxidase (NOX), enhance antioxidant activity by scavenging free radicals and inactivating their reactions [78,115]. This results in accelerated and successful healing with less time for wound closure. Oxidative stress resulting from oxidants and antioxidants imbalance disrupts redox signaling, causing molecular damage and an inability to neutralize and protect against reactive damage. This imbalance, when the ability to counter-regulate the pro-oxidant state is lost, is a central cause of oxidative stress. The successful control of inflammation and oxidative stress requires a joint effort in the pursuit of tissue repair. Thus, inflammation and oxidative stress have a necessary role in the biological healing response. The answer to this question is primarily related to inflammation. Chronically, the higher the production of pro-inflammatory or the lower the production of anti-inflammatory factors, the higher the continuous production of inflammatory products (silent inflammation) and the regulation of other important processes. Regulation can occur by modulating signaling pathways, influencing antioxidant enzyme synthesis, repair and healing processes, apoptosis, and cell proliferation in a continuous and complexly modulated cycle (Figure 6).

### 4.3. Clinical Resolution of Inflammation and Skin Repair

**Figure 7:**
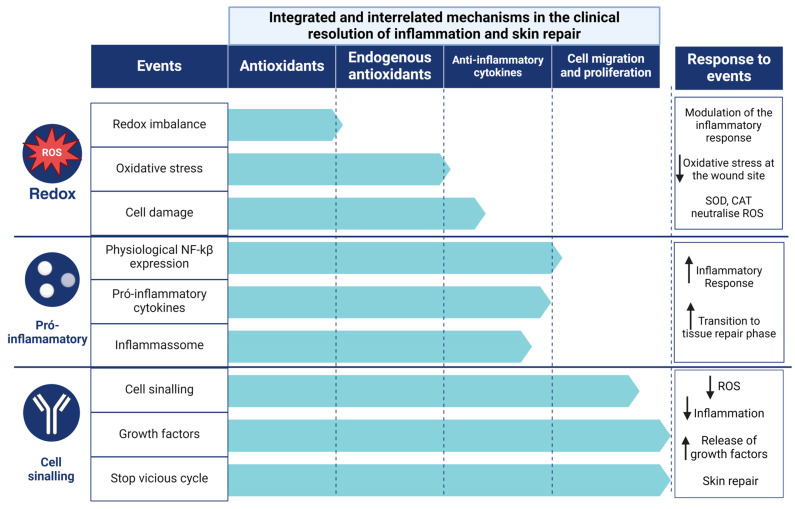
The clinical antioxidant and anti-inflammatory modulation of inflammatory mechanisms and the consequent reduction and control of oxidative inflammation.

Obesity, vascular disease, neurodegenerative disease, burns, and diabetes with ulcers [35,39,75,80,81,82,116,117,118] are the main factors contributing to chronic wounds, which account for a large proportion of healthcare costs. Complications related to inflammation contribute to delayed wound closure. One of the most important is the hypoxia response pathway [118], particularly in hyperglycemic patients, resulting in impaired neovascularisation and poor wound healing outcomes, as well as impaired cell migration and production of anti-inflammatory agents that control OxInflammation. This process can lead to secondary infections due to the colonization of microorganisms prone to biofilm formation [119,120]. Acceptable complications of delayed healing include increased risk of secondary infection, chronic pain, reduced quality of life, and potential progression to chronic ulcers. However, prompt treatment reduces chronic inflammation, promotes tissue regeneration, and prevents associated complications. This study focused on the mechanisms that influence inflammation, which can affect wound healing in different ways, regardless of wound size or delay in the onset of healing. In this context, we have identified the leading influencers in the control and resolution of inflammation and regulation of skin wound healing (Figure 7). Therefore, a hierarchical design is used to reduce the redox balance, the expression of pro-inflammatory cytokines, the activity of the inflammasome, the bacterial load and the stimulation of tissue regeneration. By identifying the therapeutic targets and levels of resolution involved in regulating the inflammatory response at a clinical level, we point to the action of antioxidants in controlling the inflammatory response over time and improving the quality of regenerated skin. Furthermore, the demonstration of a low risk of bias in the studies and the replication of the results in different experimental contexts have increased confidence in the efficacy of antioxidants as a therapy to accelerate wound healing.

### 4.4. Limitations

Systematic reviews are considered to be high-level studies, depending on the quality of the studies, and allow individual studies to be assessed in a blinded manner (without the influence of researchers), helping with decision-making, identifying gaps in knowledge, and allowing individual studies that can then guide future studies. In this case, the methodological heterogeneity between the studies analyzed was identified mainly from the divergent characteristics of the animal models, such as age, weight, and total number of animals. When assessing the risk of bias and methodological quality, we found that most of the studies did not clearly indicate the main outcomes assessed, and although only two studies (each) omitted information on wound closure and the intervention used, these studies presented a high risk of methodological bias, which prevented the reproducibility of the studies. Using the SYRCLE tool, we identified specific limitations in the research reports, mainly related to the omission of important information, such as the experimental randomization procedures, the procedures for assigning the animals to the experimental groups, the way the animals were housed, the data collection methods and the blinding of the researchers in relation to the experimental groups.

## 5. Conclusions

Our results confirm that oxidative stress and inflammation are coexisting and interrelated events. These events are involved in feedback mechanisms that ensure the reciprocal stimulation of cytokines and chemokines, membrane receptors, inflammatory signaling pathways, antioxidants, and other growth factors regulated during the inflammatory process in different diseases affecting the population, as these molecules act in different regulatory pathways and exert specific functions at each stage of the healing process, inflammatory resolution, and clinical condition. In this review, we found that IP3, IKK (IK-β and NFK-β), and IFN-δ are the most studied molecular pathways in the healing process of skin wounds. These pathways trigger the production of pro-inflammatory cytokines, such as TNF-α and IL-1, and stimulate signaling and M1 polarization through the activation of membrane receptors or cytosolic pathways. Physiologically, NF-κβ induces NLRP3, which stimulates the production of mtROS, triggering a respiratory burst that amplifies the pro-oxidative microenvironment and oxidative stress. In chronic wounds, this process becomes a vicious cycle of increased inflammation, delayed healing, and/or hypertrophic scarring that threatens resolution. Furthermore, few studies have assessed the total landscape of pro-inflammatory (IL-1, IL-6, IL-8, EGF, and ANG 1–2) and anti-inflammatory (IL-10, CRP, and fibrinogen) cytokines or chemokines, oxidative stress bioproducts (LPO, MDA, PCN, ON, 4-HNE, and DNA damage); which are important tools to understand their role in the complex dynamic equilibrium during wound healing. In general, inflammation is beneficial when acute and detrimental when prolonged. Taking this into account, therapeutic inhibition of oxidative and inflammatory events should progressively lead to inflammatory resolution and tissue regeneration by reducing the redox balance, the expression of pro-inflammatory cytokines, the activity of the inflammasome, and the bacterial load. Therefore, our results provide new insights into the relationship between oxidative stress and inflammation in the inflammatory process of wound healing.

## Data Availability

The data can be made available upon request through the email: fernanda.b.lopes@ufv.br.

## Supplementary Materials

The following are available online at https://www.mdpi.com/article/10.3390/antiox13070823/s1, Table S1. Complete search strategy with search filters and a number of studies recovered in the databases PubMed-Medline, Scopus, and Web of Science. Table S2. A description of the main features of the studies included in this systematic review that evaluated oxidative stress and inflammation in excision wound healing is included. Table S3. Description of the main features related to excisional wounds and days of wound healing monitoring.
