# Supplementary material for: OxInflammatory Responses in the Wound Healing Process: A Systematic Review
Source: Antioxidants (Basel). 2024 Jul 9;13(7):823. doi: 10.3390/antiox13070823 (PMC11274091; doi:10.3390/antiox13070823)
Supplement: Supplementary file 1 [file antioxidants-13-00823-s001.zip › antioxidants-3018954-supplementary.pdf]

Table S1: Filters

Data base	Descriptors	Items Found	Time	Date
PUBMED	#1 Filter wound healing<br>("wound healing"[MeSH Terms] OR "wound healing"[Title/Abstract])	169,760	14:42:26	13/04/2021
	#2 Filter antioxidants<br>("antioxidants"[MeSH Terms] OR "antioxidants"[Title/Abstract] OR "oxidative stresses"[Title/Abstract] OR "antioxidative stress"[Title/Abstract] OR "oxidative stress injury"[Title/Abstract])	169,099	14:42:42	13/04/2021
	# 3 Filter inflamation<br>("inflammation"[MeSH Terms] OR "inflammation mediators"[MeSH Terms])	719,904	14:57:04	13/04/2021
	Total: #1 and #2 and #3<br>("wound healing"[MeSH Terms] OR "wound healing"[Title/Abstract]) AND ("antioxidants"[MeSH Terms] OR "antioxidants"[Title/Abstract] OR "oxidative stresses"[Title/Abstract] OR "antioxidative stress"[Title/Abstract] OR "oxidative stress injury"[Title/Abstract]) AND ("inflammation"[MeSH Terms] OR "inflammation mediators"[MeSH Terms])	121	15:05:27	13/04/2021
**Data base**	**Descriptors**	**Items Found**	**Time**	**Date**
SCOPUS	#1 Filter wound healing<br>(TITLE-ABS-KEY ("wound healing"))	182,548	15:10:02	13/04/2021
	#2 Filter antioxidants<br>(TITLE-ABS-KEY ("antioxidants")) OR (TITLE-ABS-KEY ("oxidative stresses")) OR (TITLE-ABS-KEY ("antioxidative stress")) OR (TITLE-ABS-KEY ("oxidative stress injury"))	638,662	15:11:04	13/04/2021
	#3 Filter inflammation<br>(TITLE-ABS-KEY ("inflammation")) OR (TITLE-ABS-KEY (“inflammation mediators"))	809,911	15:13:03	13/04/2021
	Total: #1 and #2 and #3<br>((TITLE-ABS-KEY (“wound healing"))) AND ((TITLE-ABS-KEY ("antioxidants")) OR (TITLE-ABS-KEY ("oxidative stresses")) OR (TITLE-ABS-KEY ("antioxidative stress")) OR (TITLE-ABS-KEY ("oxidative stress injury"))) AND ((TITLE-ABS-KEY ("inflammation")) OR (TITLE-ABS-KEY ("inflammation mediators"))) AND (LIMIT-TO (EXACTKEYWORD, "Animal Model"))	383	15:16:07	13/04/2021
**Data base**	**Descriptors**	**Items Found**	**Time**	**Date**
WEB of SCIENCE	#1 Filter wound healing<br>(TS=wound healing)	89,305	15:20:08	13/04/2021
	#2 Filter antioxidants<br>(TS=antioxidants OR TS=oxidative stresses OR TS=antioxidative stress OR TS=oxidative stress injury)	649,047	15:22:39	13/04/2021
	#3 Filter inflamation<br>(TS=inflammation)	623,193	15:24:47	13/04/2021
	#4 Filter animals<br>TS=Mice OR TS=Mouse OR TS=Rat OR TS=Rats OR TS=Dog OR TS=Dogs OR TS=Rabbits OR TS=Murine model OR TS=Guinea pig OR TS=Hamster OR TS=Animal model	4.113,737	15:26:43	13/04/2021

	Total: #1 and #2 and #3 and #4<br>(TS=wound healing) AND (TS=antioxidants OR TS=oxidative stresses OR TS=antioxidative stress OR TS=oxidative stress injury) and (TS=inflammation) AND (TS=Mice OR TS=Mouse OR TS=Rat OR TS=Rats OR TS=Dog OR TS=Dogs OR TS=Rabbits OR TS=Murine model OR TS=Guinea pig OR TS=Hamster OR TS=Animal model)	541	15:28:35	13/04/2021

Table S2: Animal model – Rat and mice

Reference	*Country*	*Strain*	*Sex*	*Age*	*Weight*	*Control group*	*Ethics Committee*	*Statistical test*
Back et al., 2020	Brazil	Wistar rats	M	2 mo	?	Negative: untreated<br>Positive: Dersani®	Yes	Anova
Dhall et al., 2016	United States of America	C57BL/6 mice	?	24 weeks	?	Negative: untreated	Yes	Anova; Student's t test.
Dwivedi et al., 2017	India	Wistar rats	?	?	160 - 180 g	Negative: DMSO.<br>Positive: Vitamin E	Yes	Anova; hoc Scheffe's test.
Ganeshkumar et al., 2012	India	Wistar rats	M	?	180 - 200 g	Negative: PBS	Yes	Anova
Gangwar et al., 2015	India	Charles Foster albino rats	?	?	150 - 200 g	Negative CMC<br>Positive: Vitamin E	Yes	Anova; Dunnett's test
Gautam et al., 2014	India	Charles-Foster albino rats	M/F	?	180 - 230 g	Negative: CMC<br>Positive: Vitamin E	Yes	Anova; Dunnett's test
Jridi et al., 2017	Tunisia	Wistar rats	?	Youngs	150 - 200 g	Negative: untreated<br>Positive: Cicaflora©	Yes	Anova; Dunnett's test
Kandhare et al., 2015	India	Wistar rats	M	Adults	180-200g	Negative: Did not receive injury; No receive any drug treatment.<br>Positive: framycine sulfate ointment	Yes	Anova; Bonferroni's test
Leu et al., 2012	Taiwan	BALB c/mice	?	8 weeks	?	Negative: untreated	Yes	Student's t-test and Tukey-Kramer
Lim et al., 2006	United States of America	Hairless SKH-1 mice	F	8 weeks<br>18 months	?	Negative: Exposed to ambient filtered room air	Yes	Anova
Murthy et al., 2013	India	Charles-Foster albino rats	M/F	?	160-180g	Negative: CMC<br>Positive: Vitamin E	Yes	Anova; Dunnett's test
Nafiu & Rahman, 2014	Malaysia	Sprague–Dawley rats	F	Adults	?	Negative: Deionized water<br>Positive: Solcoseryl®	Yes	One way-Anova.
Park et al., 2010	Korea	CrljBgi: CD-1 (ICR) mice	F	4 weeks	?	Negative: Diet no CLA isomers	Yes	Anova; Dunnett's test
Park et al., 2011	Korea	ICR mice	F	4 weeks	?	Negative: untreated	Yes	Duncan's - SPSS Statistics

Patel et al., 2019	Japan	Sprague–Dawley rats	M	9 weeks	290-310g	Negative: blank hydrogels (without drugs)	Yes	Anova
Sarandy et al., 2018	Brazil	Wistar rats (Rattus norvegicus)	M	5 weeks	198,25 ± 26,11g	Negative: saline<br>Positive: silver sulfadiazine	Yes	D'Agostino-Pearson test; Kruskal-Wallis
Schanuel et al., 2020	Brazil	C57bl/6 mice	M	12 weeks	?	Negative: standard (10% energy from fat) chow	Yes	Kolmogorov–Smirnov normality test and The parametric Welch-corrected unpaired t-test and A nonparametric Mann– Whitney test.
Singh et al., 2017	Korea	ICR mice	M	8 weeks	30g	Negative: untreated<br>Positive: Vanillyl alcohol	Yes	Anova
Sungkar et al., 2020	Indonesia	Whistar rats	M	3-4 mo	150-300g	Negative: ?	Yes	Normality test (Shapiro-Wilk test) and One Way Anova.
Yadav et al., 2017	India	Albino Wistar rats	M	Adults	180-200g	Negative: untreated<br>Positive: povidone iodine ointment	Yes	Anova; Dunnett's test
Yadav et al., 2018a	India	Albino Wistar rats	M	Adults	180 ± 20 g	Negative: untreated<br>Positive: povidone iodine ointment	Yes	Anova; Dunnett's test
Yadav et al., 2018b	India	Albino Wistar rats	M	Adults	180-200g	Negative: untreated<br>Positive: povidone iodine ointment	Yes	Anova; Dunnett's test
Zhang & Gould, 2013	United States of America	Sprague–Dawley rats	M	8 weeks	?	Negative: normoxia at sea level	Yes	nQuery Advisor 7.0; Tukey's Test

Table S3: Cutaneous wounds

Reference	*Lesion*	*Site*	*Antisepsis*	*Anesthesia*	*Analgesia*	*Inst. For Biopsy*	*Wound Nº/size*	*WH (days)*
Back et al., 2020	Excision	Dorsum	?	Xylazine (90 mg/kg) and ketamine (13 mg/kg)	cetoprofen (0.5 mg/kg)	Biopsy punch	2/50mm2	0, 2, 7, and 12
Dhall et al., 2016	Excision	Dorsum	?	?	?	Biopsy punch	?/10 mm2	0, 1, 3, 5, 7, 9, 12, 14
Dwivedi et al., 2017	Excision/Incision	Dorsum	?	?	?	?	?/500 mm2	1, 4, 7, 10, 13, 16, 19,21
Ganeshkumar et al., 2012	Excision/Incision	Dorsum	?	?	?	?	1/20 mm2	0, 4, 8, 12
Gangwar et al., 2015	Excision/Incision	Dorsum	?	Pentobarbitone (35 mg/kg); Ether	?	?	?/~500 mm2	0, 4, 8, 12, 14, 16, 18, 20, 22
Gautam et al., 2014	Excision/Incision	Dorsum	?	ketamine hydrochloride (50mg/kg)	?	?	1/≈500 mm2	0, 4, 8, 12, 14, 16, 18, 20, 22, 24
Jridi et al., 2017	Excision	Thoracic region	?	ketamine (100 mg/kg body weight)	?	?	1/~150 mm2	0, 2, 4, 6, 8, 10, 12
Kandhare et al., 2015	Excision	Dorsum	?	80 mg/kg dose of ketamine	?	?	1/300mm2	0, 4, 8, 12, 16, 20
Leu et al., 2012	Excision	Dorsum	Betadine	2 to 2.5% vaporized inhaled isoflurane	?	Scissors and a scalpel	2/ 10 mm2	0, 1, 3, 5, 7
Lim et al., 2006	Excision	Dorsum	?	Isoflurane	?	Biopsy punch	2/ 3.5mm	0, 1, 2, 3, 4, 5, 6, 7, 8, 9
Murthy et al., 2013	Excision/Incision	Dorsum	?	ketamine (30mg/kg, ip)	?	?	1/ ≈500mm2	0, 4, 8, 12, 14, 16, 18, 20, 22
Nafiu & Rahman, 2014	Excision	Dorsum	?	Ethyl ether	?	Biopsy punch	?/6mm	0, 1, 3, 5, 7, 9, 11
Park et al., 2010	Excision	Dorsum	Alcohol	Isoflurane	?	Biopsy punch	2/4mm	0, 1, 2, 3
Park et al., 2011	Excision	Dorsum	?	Isoflurane	?	Biopsy punch	2/4mm	0, 1, 2, 3
Patel et al., 2019	Excision	Dorsum	Saline and ethanol	Isoflurane	?	?	1/20 x 20mm	0, 4, 8
Sarandy et al., 2018	Excision	Dorsum	?	ketamine (60 mg/kg) and xylazine (10 mg/kg)	Pentobarbital (30mg)	Biopsy punch	3/ 12 mm	0, 7, 14, 21
Schanuel et al., 2020	Excision	Dorsum	?	ketamine (150 mg/kg) and xylazine (15 mg/kg)	?	?	1/10 mm2	0, 6, 10
Singh et al., 2017	Excision	Dorsum	?	?	?	?	4/ 8mm2	3, 5, 7
Sungkar et al., 2020	Excision	Dorsum	Alcohol	?	?	?	1/ 20 mm2	?
Yadav et al., 2017	Excision/Incision	Dorsum	Saline	Ethyl ether	?	Scissors and a scalpel	1/ 500 mm2	3, 6, 9, 12, 15, 18
Yadav et al., 2018a	Excision/Incision	Dorsum	Saline	Ethyl ether	?	Scissors and a scalpel	1/ 500 mm2	0, 3, 6, 9, 12, 15, 17
Yadav et al., 2018b	Excision/Incision	Dorsum	Saline	Ethyl ether	?	Scissors and a scalpel	1/ 500 mm2	0, 3, 6, 9, 12, 16
Zhang & Gould, 2013	Excision	?	?	?	?	?	?	?
